# SP1 enhances Zbtb7A gene expression via direct binding to GC box in HePG2 cells

**DOI:** 10.1186/1756-0500-2-175

**Published:** 2009-09-02

**Authors:** Xuyu Zu, Lingling Yu, Qinsheng Sun, Feng Liu, Jue Wang, Zhenhua Xie, Ying Wang, Wei Xu, Yuyang Jiang

**Affiliations:** 1 The Key Laboratory of Chemical Biology, Guangdong Province, Graduate School at Shenzhen, Tsinghua University, Shenzhen, Guangdong 518055, PR China; 2 School of Medicine, Tsinghua University, Beijing 100084, PR China; 3 School of Life Science and Biopharmaceutics, Shenyang Pharmaceutical University, Shenyang 110016, PR China

## Abstract

**Background:**

Zbtb7A is a proto-oncogenic transcriptional regulator that plays an important role in adipogenesis, osteogenesis and oncogenesis, but little is known about the regulation of Zbtb7A gene expression which is of importance in the function uncovering of this gene.

**Finding:**

Here, a 5'-flanking region of the human Zbtb7A gene was cloned and characterized. It was found that the GC box within Zbtb7A promoter is necessary for the promoter activity. Furthermore, we identified that Sp1 acts as an activator in the regulation of Zbtb7A promoter activity and the physical interaction between Sp1 and GC box is responsible for the activation of Zbtb7A gene promoter.

**Conclusion:**

Our results confirmed that Sp1 upregulates Zbtb7A gene expression via direct binding to GC box within the promoter.

## Background

Zbtb7A (FBI-1/Pokemon), an oncogene, is involved in adipogenesis, osteogenesis and oncogenesis. Zbtb7A contains a POZ/BTB domain at the N-terminus and four Krüppel-type C2H2 zinc fingers at C-terminus [1]. POZ/BTB domain mediates homodimerization and heterodimerization, and recruitment of corepressors and HDAC complexes, whereas the C2H2 zinc fingers mediate specific DNA recognition and binding [2,3].

Zbtb7A was originally identified as a protein that binds specifically to a HIV type I promoter element [3] and can physically interact with other POK family members such as BCL-6 [4]. Zbtb7A has pleiotropic functions such as repression of genes transcription of ADH5/FDH [5], Rb [6], FANS [7], CyclinA gene and E2F4[8], NF-KB trans-activation[9], playing important role in adipogenesis[10], and osteoclastogenesis [11]. Zbtb7A is also a repressor of the ARF tumor suppressor gene (p19Arf in the mouse, and p14ARF in humans) that in turn lowers the expression of another tumor suppressor p53 gene, and is a central regulator in oncogenesis[1]. Zbtb7A gene amplification is a relatively frequent event and leads to the over expression of this gene in non-small cell lung cancer[12]. Recently it was reported that proto-oncogene Zbtb7A and SREBP-1 synergistically activate transcription of fatty acid synthase gene(FASN)[7].

The mechanisms of Zbtb7A gene expression regulation is of importance for understanding the precise roles of Zbtb7A in physiological and pathological processes. To date little is known about the transcriptional regulation of the Zbtb7A gene.

To better understand the molecular mechanism regulating expression of the Zbtb7A gene in humans, 5'-upstream region of Zbtb7A gene was cloned and analyzed. We confirmed that the proximal 1000 bp upstream of the translation start site mediates most of the basal activity and contains two Sp1 binding sites, and we for the first time provide evidence that Sp1 enhances the transcription of Zbtb7Agene through direct binding to GC box of the promoter.

## Methods

### Cell line and cell culture

Human liver cancer HepG-2 cells and human embryo kidney 293-T cells (American TypeCulture Collection, Manassas, VA) were cultured under DMEM medium containing 10% fetal bovine serum(Hyclone, USA), L-glutamine(2 mM), streptomycin(0.1 mg/ml) and penicillin(100 U/ml) at 37°C in a humidified incubator supplied with 5% CO_2_.

### Plasmids preparations

Various 5'-3' deletion constructs of pokemon, 4000 bp, 2000 bp, 1000 bp, and 500 bp DNA fragment relative to a putative transcriptional start site, were generated from HepG-2 cells. For construction of the putative Zbtb7A promoter deletion constructs, the reverse primer for PCR is 5'-GACAAGCTTCTTCCGCGCCGAGACC-3', and the forward primers were follow:5'-GTAAGATCTGAGGAACGGCCCAGCAG-3'(4000 bp),5'-GCTAGATCTGTAGCTGGGATTACAGGCACG-3'(2000 bp), AAATTGAGGCTGGGG-3'(1000 bp),5'-GCTAGATCTTCATGCCATTCTCCTGCCTC-3'(500 bp). PCR-amplified products were digested with enzymes Bglα and Hindβ, and cloned into the corresponding sites of the pGl4.10-basic Firely Luciferase expression plasmid (Promega) to generate the plasmids pLuc-4000, pLuc-2000, pLuc-1000, pLuc-500 respectively. The internal control pRL-TK Renilla Luciferase vector was purchased from Promega Corp. The Sp1 expression construct was generated by inserting a cDNA fragment obtained by reverse transcription PCR from HepG-2cell into pcDNA3.1 (-) vector at the HindIII and EcoRI sites. The PCR oligonucleotide primer pair used for the Sp1 cDNA amplification is as follow: 5'-GAAGAATTCCATGAGCGACCAAGATCAC-3' and reverse 5'-GAAAAGCTTCTCTTGGACCCATGCTAC-3'.

### Site directed mutagenesis

Sp1 putative recognition sites mutation of Zbtb7Apromoter were obtained by preparing the pLuc638m and pLuc969m constructs, using mutated primers within GC-boxes. The plasmids with mutation was generated by using site-directed mutagenesis system (Promega) according to the protocol recommended by manufacture and pLuc1000 construct was used as a template. The oligonucleotides used for mutagenesis (mutations indicated with inclined form and bold letters) are following: pLuc638m, 5'-AATGATCC***AAAAAAAA***CTGCCTCCCAAG-3'; pLuc969m, 5'- CCCATCTGTACAC***AAAAAAAA***CCAGCTGTC-3'.

DNA sequencing was performed to confirm that the sequence of the PCR products were correct as compared with the Zbtb7Apromoter published in the Human Genome database.

### Transient Transfections, Luciferase Assay

1 × 10^5 ^293 T and HepG-2 cells were seeded respectively into 24 wells, and 2 μl of Lipofectamine-2000(Invitrogen, CA, USA) was used for plasmids transfection. At 48 h After transfection, the cells were harvested, lysed, and luciferase activities were measured in triplicate using Dual Luciferase™ Reporter Assay System (Promega, Wallisellen, Switzerland).

### RT-PCR

Total RNA was prepared from HepG-2 cells using TRIZOL reagent (Invitrogen, CA), and cDNA was synthesized using a reverse transcription synthesis system (TOYOBO, Japan), according to the manufacture's recommendations. Oligonucleotide primers used for PCR are as follow: pokemon, forward, 5'- GAAGCCCTACGAGTGCAACATC-3', reverse 5'- GTGGTTCTTCAGGTCGTAGTTGTG-3'; Sp1, forward: CATGAGCGAC CAAGATCAC, reverse: CTCTTGGACCCATGCTAC; GAPDH, forward: 5'- CAACGTGTCAGTGGTGGACCTG-3', reverse: 5'- TTACTCCTTGGAGGCCATGTGG-3'.

### Chromatin immunoprecipitation (ChIP) Assay

ChIP Assay was carried out to analyze the physical interaction between Sp1 and Zbtb7Apromoter in the HepG-2 cells. Formaldehyde was added at 1% to the culture media for 10 min at 37°C to cross-link protein to DNA. Cells were washed twice with ice-cold 1 × PBS, scraped, and resuspended in ChIP sonication buffer (1% Triton-100, 0.1% Deoxycholate, 50 mM Tris 8.1, 150 mM NaCl, 5 mM EDTA) containing protease inhibitors. The cells were sonicated to shear DNA to lengths between 2000 bp to 500 bp. The sonicated supernatant was diluted with ChIP dilution buffer, and incubated with antibody against Sp1(Abcam) overnight at 4°C with rotation. The chromatin-antibody complexes were collected by Dynabeads protein G (Invitrogen). After wash with pH 5.0 TE beffer (10 mM Tris, 1 mM EDTA) for three times, the pellet was dissolved with pH 3.0 TE buffer. After precipitation with ethanol, the pellets were resuspended and treated with proteinase K. The supernatant was extracted with saturated NaCl and precipitated with 2-propanol to recover DNA. The primers for ChIP PCR were designed to amplify two putative Sp1 binding sites in Zbtb7Acore promoter sequence: primer forward, 5'- GGGAAACTGAGGCTGATGG-3', reverse, 5'- TCAGGTGTCCCACTCCCAAC-3' for-1003 bp to -925 bp amplification, and primers forward, 5'- ACTGCACTTGGGAACAGC-3', reverse, 5'-GGCAACAGAGCAAGACTC-3' for -774 bp to -584 bp amplification.

## Results

### Cloning and characterization of the 5' regulatory region of Zbtb7Agene

**Figure 1:**
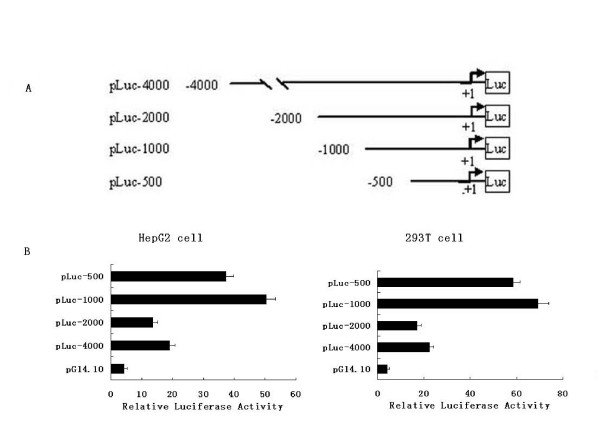
**Deletion and mutation analysis of the Zbtb7Acore promoter activity**. A, schematic representation of Zbtb7Apromoter luciferase report plasmids: pLuc-4000, pLuc-2000, pLuc-1000, pLuc-500, containing a series of 5'-3'deleted promoters; B, C, HepG-2 and 293 T cells were transient tranfected with 0.8 ug of either Zbtb7Apromoter plasmids or control plasmid (pGl4.10).

To identify potential cis-acting elements required for basal Zbtb7Aactivity, we cloned a 4000 bp of DNA fragment from -4000 to +1, relative to a putative transcriptional start site from HePG-2 cells. To determine the probable promoter activity region of the Zbtb7Agene, we generated a series of 5'-3' deleted luciferase reporter constructs, containing 4000, 2000, 1000, and 500 bp fragments of Zbtb7Apromoters (Fig. 1A). Functional analysis of those constructs showed that a proximal promoter (-1000/+1) maintains high level of basal activity in both 293 T and HePG2 cells (Fig 1B and Fig 1C), which suggested that it would contain some important cis-acting elements.

**Figure 2:**
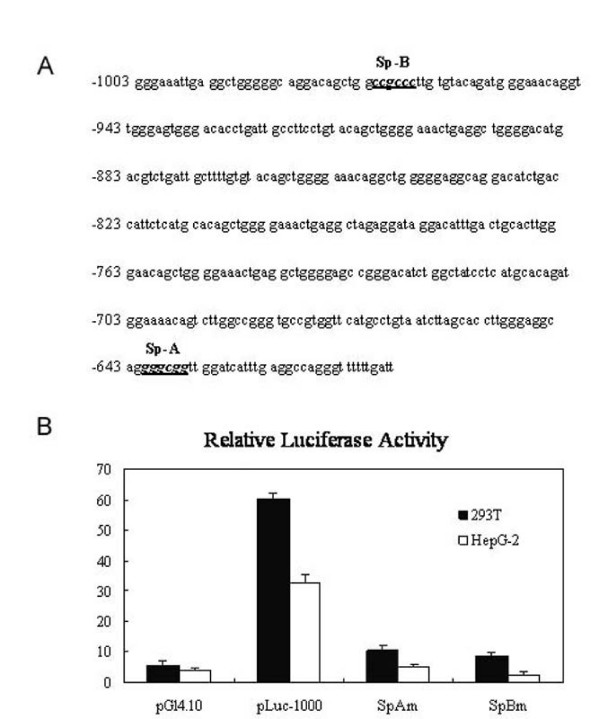
**Searching for potential cis-elements in the 1000 bp Zbtb7Apromoter**. A, The core promoter contains consensus sequences for binding transcription factor Sp1(bold and underlined); B, Mutation analysis of the Sp1-binding sites in the Zbtb7Apromoter. Two mutants were constructed and used for transient promoter assays.

We made use of TFSEARCH program  to search for potential cis-elements in the 1000 bp Zbtb7Apromoter, and found two putative Sp1 binding sites: SpA(-641~636-) and SpB(-972~-967) as shown in Fig 2A. To determined whether both putative elements were responsible for basal activity of the Zbtb7Agene, we mutated those two GC-boxes (also Sp1 binding site)[13]. As shown in Fig 2B, the mutation of *GGGCGG *to *AAAAAA *of SpA and SpB binding sites resulted in a 6.3-fold and 14.6-folds decrease of luciferase activity in HepG2 cells and 5.6-folds and 7.2 folds decrease in 293 T cells respectively. These results revealed that two GC-boxes binding sites are required for core promoter activity of the Zbtb7Agene.

### Sp1 enhances the core promoter activity of Zbtb7A gene

**Figure 3:**
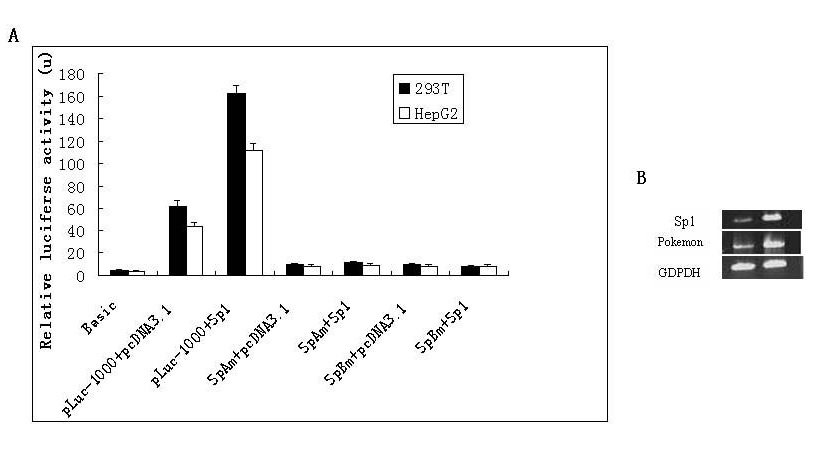
**Sp1 increased basal promoter activity could be abrogated by the GC boxes mutation**. A, Sp1 enhances the core promoter activity of Zbtb7Agene. HepG-2 and 293 T cells were transient co-tranfected with either pLuc-1000 and pCDNA3.1, or Sp1 and pLuc-10000 or SpAm or SpBm. pGl4.10 was control B, RT-PCR analysis of Zbtb7AmRNA using the total RNA was isolated from 293 T cells tranfected Sp1.GAPDH was control. Data for A means for ± SD from three independent experiments, with each experiment carried out in triplicate.

To further understand the role of Sp1 in Zbtb7Agene transcription, we used 293 T and HePG2 cells as cell model for luciferase reporter assay. Data in Fig. 3A showed that core promoter activity of the Zbtb7Agene increased with the elevated amount of Sp1 expression constructs in 293 T and HePG2 cells, which indicates that basal promoter activity of Zbtb7Agene could be enhanced by the expression of Sp1. Whereas the Sp1 increased basal promoter activity could be abrogated by the mutation of GC boxes which suggestes that the *GGGCGG *sequence contributes to Sp1 induced activation of basal promoter activity of Zbtb7Agene (Fig. 3A). RT-PCR was used to confirm those results, and as shown in Fig. 3B, Zbtb mRNA was greatly elevated by the over expression of Sp1 in 293 T cells.

### Sp1 directly binds to the proximal promoter of pokemon

**Figure 4:**
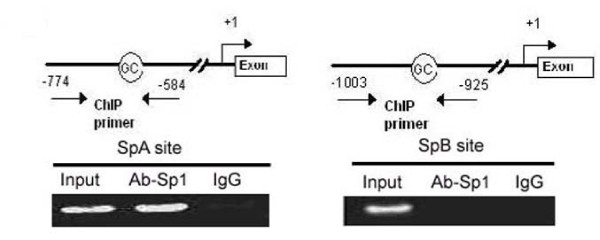
**Chromaitin immunoprecipitation (ChIP) assays**. Sheared chromatin from HePG2 cells was immunoprecipitated using Sp1 antibody. PCR primers were designed to amplify sequences:-1003 to -925(SpB site) and -774 to -584(SpA site) in the core promoter of Zbtb7Agene.

To further investigate the role of the GC-boxes in regulating the Zbtb7Apromoter activity in response to Sp1, we performed ChIP analysis to evaluate the binding capacity of Sp1 to bind Zbtb7Apromoter. Two pairs of primers were designed corresponding to SpA and SpB sites for the ChIP PCR. As shown in Fig 4, Sp1 is capable of binding to SpA site, whereas SpB site dose not show direct physical interaction with Sp1.

## Discussion

Sp1 (specificity protein 1), an important transcription factor, is ubiquitous expressed and has three C_2_H_2_-type zinc figures as DNA binding domain(DBD) [13-18]. Sp1 bind with higher affinity to GC boxes than to GT boxes or CT boxes and Sp1 bind to GC-boxes with the consensus sequence 5'-GGGCGG-3' or 5'-GGCG-3'[19,20]. An important role of Sp1 in cell growth control and tumorigenesis was reported recently [21-26]. Many Sp1 targeting genes, such as CyclinE, Cdk2, E2F1 and c-Myc, are key regulator of cell proliferation and carcinogenesis [27,28].

Zbtb7A was first identified as a cellular factor binding to a specific sequence within the human immunodeficiency virus, type 1 promoter[3]. A serial of reports revealed important roles of Zbtb7A in human and mouse adipogenesis [7], human cancer pathogenesis [1,9] and cell's determination of B versus T lineage [29]. Zbtb7Afunctions as an active regulator of genes expression, and several tumor repressors such as Rb and ARF can be repressed by Zbtb7A[1]. Although its importance in pathology and physiology, the Zbtb7Aexpression regulation remains elusive.

The results in the present study identify important cis-elements located in the proximal Zbtb7Apromoter (-1000-+1) which are responsible for basal transcriptional activity of the promoter. Two GC boxes, the core consensus of Sp1 binding site, were identified, and the mutation of Sp-A and Sp-B leaded to an approximate 80% decrease of the promoter basal activity respectively in both 293 T and HePG2 cells, indicating the importance of those sites in maintaining Zbtb7A promoter basal activity. ChIP analysis revealed that Sp1 could physically interact with Sp-A site, whereas the site of Sp-B showed no direct interaction with Sp1. The fail of Sp-B binding with Sp1 may be due to the involvement of other cofactors in the Sp-B site, since it was reported that other Sp family protein such as Sp3 also have high affinity with GC box[23]. Toyoda A et al revealed that only three of five GC box sequences in human NADH-cytochrome b5 reductase-encoding gene (CYTB5R) promoter are Sp1 responsive elements which suggest Sp1-mediated regulation of gene transcription occurs in a promoter context-dependent manner[30]. Furthermore, luciferase activity assay showed that the basal activity of Zbtb7A promoter could be activated by the Sp1 and Sp1 can also up-regulate the transcription of pokmon mRNA which indicates that Sp1 acts as a positive regulator in the expression of pokmon gene.

In summary, in this study, we for the first time provide evidence that Sp1 contributes to the Zbtb7Aexpression regulation through direct binding to the GC-rich element within the promoter region.

## Competing interests

The authors declare that they have no competing interests.

## Authors' contributions

YJ and XZ conceived the idea for this work. LY, XZ, QS, FL, JW, ZX, and YW performed the laboratory analyses. YJ, XZ and WX drafted the manuscript. All authors read and approved the final manuscript.
